# Emergence of plasmid stability under non-selective conditions maintains antibiotic resistance

**DOI:** 10.1038/s41467-019-10600-7

**Published:** 2019-06-13

**Authors:** Tanita Wein, Nils F. Hülter, Itzhak Mizrahi, Tal Dagan

**Affiliations:** 1 Institute of Microbiology, Kiel University, 24118 Kiel, Germany; 2 The Department of Life Sciences & The National Institute for Biotechnology in the Negev, Ben-Gurion University of the Negev, Beer-Sheva, 84105 Israel

**Keywords:** Experimental evolution, Molecular evolution, Antimicrobial resistance

## Abstract

Plasmid acquisition is an important mechanism of rapid adaptation and niche expansion in prokaryotes. Positive selection for plasmid-coded functions is a major driver of plasmid evolution, while plasmids that do not confer a selective advantage are considered costly and expected to go extinct. Yet, plasmids are ubiquitous in nature, and their persistence remains an evolutionary paradox. Here, we demonstrate that non-mobile plasmids persist over evolutionary timescales without selection for the plasmid function. Evolving a minimal plasmid encoding for antibiotics resistance in *Escherichia coli*, we discover that plasmid stability emerges in the absence of antibiotics and that plasmid loss is determined by transcription-replication conflicts. We further find that environmental conditions modulate these conflicts and plasmid persistence. Silencing the transcription of the resistance gene results in stable plasmids that become fixed in the population. Evolution of plasmid stability under non-selective conditions provides an evolutionary explanation for the ubiquity of plasmids in nature.

## Abstract (web summary)

It is expected that plasmids are costly and therefore that selection is required to maintain them within bacterial populations. Here, Wein et al. show that plasmid stability can emerge even in the absence of positive selection and that loss may be determined by transcription-replication conflict.

## Introduction

Plasmids play a major role in microbial ecology and evolution as vehicles of lateral gene transfer and reservoirs of accessory gene functions in microbial populations. Examples are the contribution of plasmids to rapid adaptation in growth limiting conditions (e.g., in the presence of antibiotics or pesticides^1–3^) or to long-term transitions in bacterial lifestyle (e.g., pathogenicity^4^ or anoxygenic photosynthesis^5^). Plasmid functions can benefit their bacterial hosts, nevertheless, plasmid replication and gene expression utilizing the host machinery are considered a metabolic burden^6^. Consequently, in the absence of selection for plasmid-encoded traits, plasmid-free cells outcompete plasmid-carrying cells, which leads to plasmid extinction^7^. Nonetheless, despite the predicted risk of extinction, plasmids are widespread in nature. The paradox of plasmid persistence remains an evolutionary dilemma^8^, that led to several theories and theoretical models aiming to explain their long-term persistence in microbial communities^9–12^.

Plasmid persistence in a population depends on stable plasmid inheritance (i.e., plasmid stability). Plasmid replication control mechanisms are important determinants of plasmid inheritance as they ensure the presence of sufficient plasmid copies in the cell prior to cell division. The segregation of low-copy plasmids during cell division typically relies on partition systems that actively distribute plasmid copies into the daughter cells. In the absence of a partition system, successful plasmid segregation depends on the multi-copy state and the physical distribution of the plasmids inside the cell^13–15^. Nonetheless, plasmid diffusion and localization may be affected by transcription dynamics of plasmid encoded genes^16^ or plasmid multimer formation^17^. The formation of plasmid multimers can lead to a decrease in the number of heritable units during cell division, hence reducing the chance of successful segregation and consequently also the plasmid persistence over time^18,19^. Mobilizable and self-transmissible plasmids are horizontally transferred, thus their persistence in the population can be facilitated by infectious transmission that leads to stable existence of the plasmid^20^. However, only about half of the currently described plasmids are mobile or self-transmissible^21^. In addition to the known plasmid stability mechanisms, the long-term persistence of plasmids is thought to largely depend on their effect on the host fitness.

Several experimental evolution studies demonstrated that imposing selection for the plasmid presence often leads to compensatory evolution of the plasmid or host chromosome that ameliorates the plasmid fitness cost and facilitates the plasmid persistence^22–30^. Thus, plasmid persistence may increase following adaptive evolution of the host or the plasmid. Nonetheless, the long-term plasmid persistence in an adapted host depends on the fitness effect of the compensatory mutations in the population, which may be disfavored in the absence of selection for the plasmid-encoded trait. For example, in *Pseudomonas* species mutations in the *gacS/A* system can alleviate plasmid fitness costs. However, *gacS/A* is a conserved global regulatory system that controls various processes in the cell, hence the fitness effect of *gacS/A* genetic variants may depend upon the environmental conditions^31^. Thus, mutations in *gacA/S* can potentially impose negative effects on the host, which would lead to plasmid loss under conditions that are not selective for plasmid maintenance^32–34^. Furthermore, many natural plasmids are considered cryptic as they do not encode for any known beneficial traits (e.g., refs. ^35,36^). For example, a recent survey of plasmids in the rat cecum microbiome revealed hundreds of small novel cryptic plasmids that seem to only encode their replication machinery, yet are stably maintained in the environment^37^. Thus, co-adaptation of plasmids and their hosts following strong selection for the plasmid presence may not always supply a sufficient explanation for the long-term plasmid persistence and abundance in nature.

In addition to plasmid–host interactions, abiotic factors that affect the host physiology and evolution should be considered as factors influencing plasmid persistence, especially in the absence of selection for the plasmid presence. For example, growth temperature has an impact on multiple cellular processes, affecting enzyme kinetics, diffusion rates as well as interactions of DNA, RNA, and proteins. Thus, the environmental temperature is likely to have consequences for plasmid replication and segregation dynamics. Additionally, fluctuating environmental conditions lead to population size variation that is a known determinant of bacterial evolution. Environmental conditions that lead to strong population bottlenecks increase the impact of random genetic drift, which may lead to a decrease in genetic variation and the probability of beneficial mutation fixation (reviewed in ref. ^38^). Similarly, population bottlenecks are likely to affect the frequency of hosts in a mixed population of hosts and non-host and by that influence the plasmid fate. Plasmid persistence under various environmental conditions and the effect of population size on plasmid evolution remains understudied.

We hypothesize that plasmid persistence in the absence of positive selection is determined by various factors including plasmid stability, host fitness, and environmental conditions. To evaluate the contribution of those different factors we conduct an evolution experiment in which we follow the persistence of a non-mobile model plasmid encoding for antibiotic resistance in *Escherichia coli* in the absence of antibiotics. To examine the effect of host environment (i.e., host physiology) on plasmid persistence we evolve our model plasmid at two growth temperatures. In addition, we test the effect of fluctuating host population size on plasmid evolution by applying different levels of population bottlenecks. Following the plasmid over time reveals instances of plasmid stability evolution without the exposure to positive selection.

## Results

### Evolution of a model plasmid under non-selective conditions

To study plasmid evolution, we constructed a minimal, non-mobile plasmid and introduced it to a naive *E. coli* K12-strain MG1655 host population. Our model plasmid pCON (2.6 kb) encodes the pBBR1 replication protein *rep*, the origin of replication (*oriV*)^39^ and *nptII* (Km^r^) under control of its native promoter^40^. The plasmid has no active partition or multimer resolution system and is of multi-copy state. To explore the long-term pCON persistence in the absence of selection for plasmid maintenance we performed an evolution experiment. In the experiment we tested for the influence of environmental temperature and population size on plasmid persistence. This included two different growth temperatures: the optimal growth temperature of *E. coli* at 37 °C and cold temperature of 20 °C, which is considered a stress inducing condition. To test the effect of population size on plasmid persistence, the populations were serially propagated using different population bottleneck sizes that were applied upon every transfer with three dilution levels: (1) 1:100 (10^7^ cells, large (L) population size), (2) 1:1000 (10^6^ cells, medium (M) population size), and (3) 1:10,000 (10^5^ cells, small (S) population size). Eight replicate populations were used as ancestors in both temperatures and population size treatments.

To determine whether the plasmid had an effect on the host fitness, we performed pairwise competition experiments^41^ between the plasmid-carrying strain and a marked wildtype strain (*E. coli* MG1655 Tm^r^) with eight replicate populations in both temperature regimes. Note that the chromosomal marker gene had no detectable fitness effect on the wildtype strain (Supplementary Fig. 1a). The competition experiments with the plasmid-carrying host strains revealed that pCON had no measurable effect on host fitness in neither of the temperatures (37 °C: *H*_0_:*w* ≥ 1, *P* = 0.0147 using Wilcoxon test, *n* = 64; 20 °C: *H*_0_:*w* ≥ 1, *P* = 0.00962 using Wilcoxon test, *n* = 32; Supplementary Fig. 1b).

### pCON persistence is higher at 20 °C in comparison to 37 °C

**Fig. 1:**
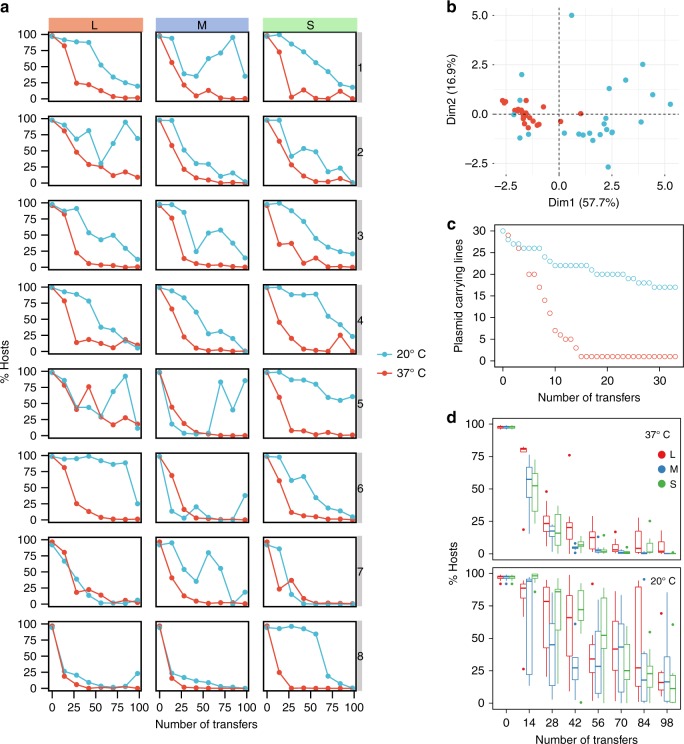
Evolution of pCON plasmid under non-selective conditions. **a** pCON persistence is shown as the proportion of hosts during the evolution experiment at 20 °C (blue) and 37 °C (red). The columns correspond to the three population bottleneck sizes (L, M, and S). The experiment was conducted for 98 transfers, which correspond to approximately 600 generations in L populations, 800 generations in M populations, and 1000 generations in S populations. To account for differential growth dynamics, the 37 °C cultures were transferred every 12 h while the 20 °C cultures were transferred every 24 h. The eight replicate lines are presented in vertical order (i.e., populations in each row have a common ancestral population). **b** Principal component analysis (PCA) of the pCON persistence over time comparing the host dynamics among the temperature regimes. **c** pCON persistence in the single colony transfer experiment at 20 °C (blue) and 37 °C (red) over a time of 33 transfers corresponding to about 800 generations. **d** A comparison of variability among pooled replicated lines (for PCA see Supplementary Fig. 3). The evolved lines are pooled according to population bottleneck size. Top: lines evolved at 37 °C. Bottom: lines evolved at 20 °C. The center value of the boxplots represents the median, the boxes denote the interquartile range, and the whiskers represent minimum and maximum values. Source data are provided as a Source Data file

The results of the evolution experiment show that, despite the lack of a fitness cost, the frequency of pCON hosts (i.e., plasmid-carrying cells) monotonously decreased over time in all replicate lines and all population sizes at 37 °C (Fig. 1a). The frequency of pCON hosts cultured in 20 °C decreased but overall slower in comparison to the host dynamics observed at 37 °C (Fig. 1a). This indicated that the plasmid loss was lower in the populations cultured at 20 °C in comparison to those cultured 37 °C. Plasmid loss occurs during plasmid segregation and can be quantified along one growth phase of the populations by measuring the appearance of plasmid-free cells^42^. We note that since pCON does not exert a significant negative impact on the host growth in either of the temperatures (Fig. 1b), the abundance of plasmid-free cells can be considered as a reliable proxy for plasmid loss frequency^43^. Nonetheless, since we do not account for the proliferation of plasmid-free cells, our plasmid loss frequency reflects the plasmid loss from the total population. Indeed, a quantification of pCON loss from the population after one growth cycle revealed a plasmid loss frequency of 4 ± 1.0% (SE, *n* = 24) plasmid-free cells at 20 °C. In contrast, at 37 °C we observed a loss frequency of 9 ± 1.1% (SE, *n* = 24) plasmid-free cells, which is significantly higher than the loss frequency at 20 °C (*P* = 0.007, *H*_0_:*f*_20°C_>*f*_37 °C_, using paired Wilcoxon test and FDR). Hence, the pCON loss over time in the evolution experiment at 37 °C was largely determined by the ancestral plasmid loss frequency, while at 20 °C the decrease in host frequency was more stochastic among replicates. Furthermore, the pCON host dynamics at 20 °C are characterized by a larger variability among replicate populations in comparison to the dynamics observed at 37 °C (Fig. 1b).

To further compare the plasmid loss dynamics in both temperatures we conducted a single colony transfer experiment under non-selective conditions. In such experiments the selection pressure is minimized and random genetic drift is maximized^44^. Thus, plasmid persistence can be attributed to plasmid stability regardless of population dynamics (e.g., genetic hitchhiking with chromosomal mutations^9^). The single colony transfer experiment was performed with 30 pCON replicate lines at 20 and 37 °C (Fig. 1c). At the end of the experiment (33 transfers) the plasmid was present in a single line at 37 °C, whereas 17 of the lines maintained the plasmid at 20 °C (Fig. 1c). This result demonstrates that pCON is more stable at 20 °C regardless of population dynamics, hence the environmental temperature had a strong influence on pCON persistence.

According to our expectation, the plasmid host frequency in a mixed population of plasmid hosts and non-hosts may depend upon the population bottleneck size. Indeed, during the early transfers at 37 °C (<14 transfers, Fig. 1d) the plasmid host dynamics appeared different among the population bottleneck treatments where the host frequency was higher in the L populations than in the M or S populations. To quantify the plasmid loss rate, we fitted a regression to the host frequency dynamics. This revealed that the plasmid loss rate over the duration of the experiment at 37 °C was 4% in the L population, 7% in the M and 5% in the S population (using linear regression of log-transformed values; Supplementary Fig. 2 and Supplementary Table 1). The estimated plasmid loss rate was significantly smaller for the L populations in comparison to the M populations (*P* = 0.0002, using *t*-test and FDR). The loss rate of S populations was marginally different from that of M populations (*P* = 0.051, using *t*-test and FDR) and was not significantly different from that of L populations (*P* = 0.232, using *t*-test and FDR). The host frequencies observed in populations evolved under the same population bottleneck at 20 °C were heterogeneous (Fig. 1d). Furthermore, the temporal pattern observed for the host frequency dynamics at 37 °C was absent at 20 °C (Supplementary Table 1). This shows that population bottleneck size had a small effect on pCON persistence at 20 °C (Fig. 1d).

### Evolution of pCON stability following a segmental duplication

Our results show that the plasmid pCON is lost over time; assuming a constant pCON loss rate at 37 °C the plasmid is expected to go extinct (Supplementary Table 1). However, plasmid host proportions stabilized after ~50 transfers in several populations (e.g., S6, S7, M6, Fig. 1a). A further examination of the host frequency at the end of the experiment revealed that pCON only went extinct in one population (S8). The discrepancy between prediction and plasmid persistence over time suggests that plasmid stability evolved in our experiment. In order to test for pCON stability, the evolved populations were transferred into plasmid-selective conditions for overnight growth. Subsequently, the resulting plasmid hosts were inoculated as the ancestral populations for a follow-up experiment under non-selective conditions.

**Fig. 2:**
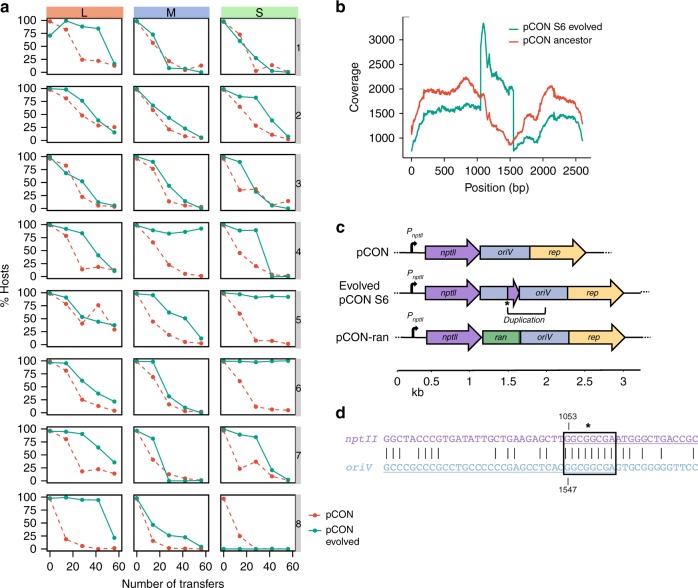
Follow-up evolution experiment of evolved pCON hosts at 37 °C. **a** Plasmid persistence under non-selective conditions after selection for evolved plasmid-carrying populations (green) compared against pCON persistence during the first experiment (Fig. 1a) at 37 °C (red, dashed lines). The experiment was conducted for 56 transfers corresponding to about 340 generations in L populations, 450 generations in M populations, and 560 generations in S populations. **b** Sequencing coverage distribution of ancestral and evolved pCON variants from population S6. The presented coverage was divided by the mean coverage. **c** Genomic map of pCON plasmids. The pCON variant S6 encountered a segmental duplication downstream the *nptII* gene including 125 bp of *nptII* and 372 bp of the *oriV*. The fusion site is indicated by a star symbol. In pCON-ran a random DNA segment of 500 bp was placed between *nptII* and the *oriV*. **d** Nucleotide sequence of the fusion site of *nptII* and the *oriV*. The black box indicates a microhomology region (8 bp) within which the illegitimate fusion potentially occurred. Extended homologies are shown up and downstream of the fusion site. Source data are provided as a Source Data file

The plasmid loss dynamics in most of the host populations were similar to the ones observed in the first evolution experiment (Fig. 2a). Nonetheless, the host frequency in some populations remained stable over time (M4, S5, and S6). To further validate this finding, we quantified the overnight plasmid loss frequency for each of the stable host populations, which revealed a very low plasmid loss (M4: 1.3 ± 0.8%, S5: 0.3 ± 0.3%, S6: 0.1 ± 0.1%, SE, *n* = 6 for each population, Supplementary Fig. 4). The evolution of plasmid stability can be attributed to genetic adaptation of either the plasmid or the host chromosome. To test for adaptation, we extracted the evolved stable plasmids and transformed them into the naive *E. coli* ancestor. Plasmids from two populations exhibited a high loss frequency in the naive host: 13 ± 3% (SE, *n* = 6; M4) and 5 ± 2% (SE, *n* = 6; S5) plasmid-free cells after overnight growth (Supplementary Fig. 4), which were not significantly different from that of the ancestral pCON population (*P*_M4_ = 0.07, *P*_M5_ = 0.69, using Wilcoxon test, *n* = 6). Thus, the evolved plasmids from population M4 and S5 did not adapt to their host. Consequently, plasmid stability in the evolved M4 and S5 populations was attributed to host adaptation (similarly to previous observations, e.g., ref. ^26^). In contrast, the overnight loss frequency of the evolved S6 plasmid in the naive host was significantly lower than the ancestral loss frequency (0.6 ± 0.3% SE, *n* = 6, *P* = 0.024, using Wilcoxon test) and not significantly different in comparison to the evolved host (*n* = 6, *P* = 0.962, using Wilcoxon test, Supplementary Fig. 4). Thus, the observed plasmid stability in the S6 population resulted from plasmid adaptation. Our results hence demonstrate that plasmid stability can evolve under conditions that are not selective for plasmid maintenance.

The observed plasmid stability in the evolved S6 population was expected to manifest in genetic modification of the plasmid. Accordingly, we sequenced the plasmids from evolved and ancestral S6 populations (Supplementary Table 2). This revealed a segmental duplication of a 497 bp fragment comprising the 3′-end of *nptII* and 372 bp of the adjacent *oriV* region in the evolved plasmid (Fig. 2b, c; note that pCON does not encode a transcription terminator after *nptII*). The sequence analysis suggested that the insertion occurred inside the *oriV* region through a recombination event that was facilitated at a microhomologous segment shared between *nptII* and the *oriV* region (Fig. 2d and Supplementary Fig. 5). To test if the duplication conferred plasmid stability, we cloned the duplicated segment into the ancestral plasmid (pCON-dup). The loss frequency of pCON-dup was similar to the evolved S6 population (0.1 ± 0.1%, SE, *n* = 6 plasmid-free cells), and confirmed that the insertion indeed conferred the observed plasmid stability. To examine whether plasmid stability was related to characteristics of the duplicated sequence, we cloned a random DNA fragment of 500 bp between the 3′-end of *nptII* and the *oriV* (pCON-ran, Fig. 2c). The loss frequency of pCON-ran was not significantly different from pCON-dup (0.3 ± 0.1%, SE, *n* = 6, *P* = 0.112 using Wilcoxon test) showing that the position of the insertion between 3′-end of *nptII* and the adjacent *oriV* region, and not the sequence characteristics, was important for plasmid stability. This implies that the position of the duplication and proximity of the *oriV* (i.e., replication initiation) to the plasmid encoded gene *nptII* (i.e., transcription) conferred the observed stability.

### Transcription, replication, and DNA topology lead to pCON instability

Conflicts between transcription and replication are a known determinant of DNA instability^45–47^ as the two processes are known to produce torsional stress ahead of their moving direction (see the twin domain model^48^). The genome architecture of the evolved pCON variant suggests that such conflicts might play a role in pCON stability. Furthermore, the coordination of transcription and replication may have been an important factor for the increased pCON stability observed in the evolutionary experiment performed at 20 °C (Fig. 1). Consequently, we compared the plasmid copy number (as a proxy for replication frequency) and the plasmid-encoded gene transcription level (*rep* and *nptII*) between instable and stable pCON plasmids in both temperature regimes.

**Fig. 3:**
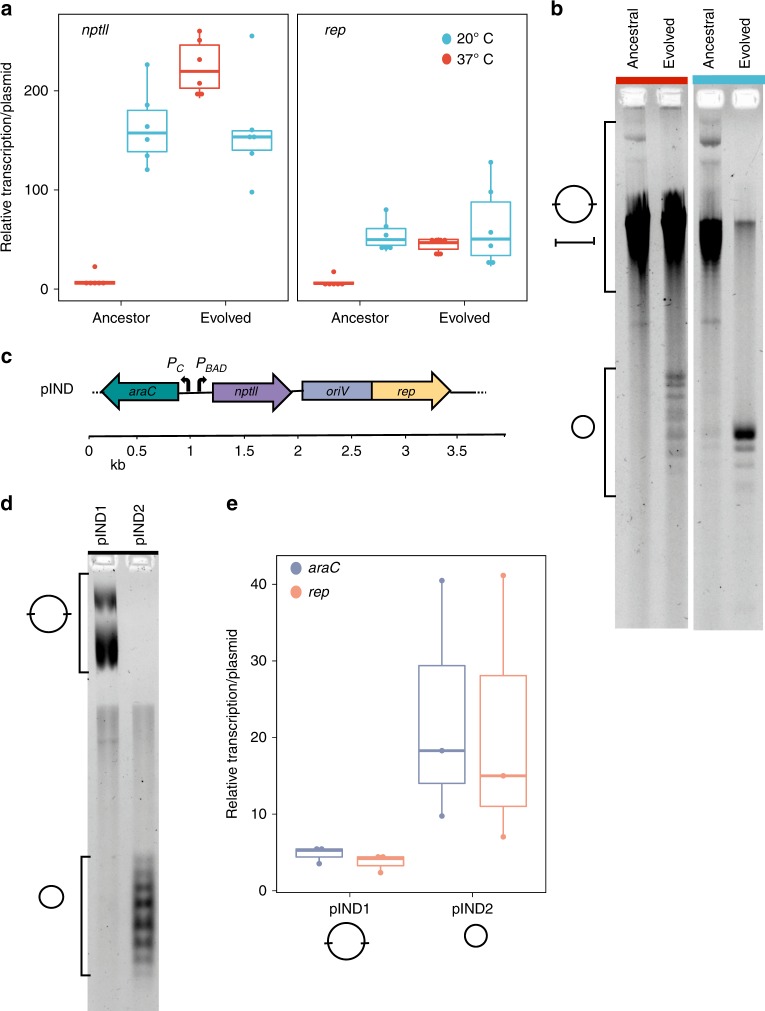
pCON transcription, replication and DNA topology. **a** Relative transcription of plasmid genes (*nptII* and *rep*) in ancestral and evolved plasmids from both temperatures was calculated per plasmid (i.e., dividing by the PCN) (Supplementary Fig. 6, *n* = 6). The evolved plasmids at 37 °C comprise the stably inherited pCON variant from population S6. **b** Separation of pCON plasmid preparations by one-dimensional agarose gel electrophoresis in the presence of chloroquine (4 µg/ml; for original gel see Supplementary Fig. 7). Chloroquine was used to resolve different topological forms of supercoiled plasmids and compare their relative motility to each other. The isolated plasmids correspond to the plasmids presented in (**a**). Symbols indicate supercoiled plasmid topoisomers of multimers (large circle) and monomers (small circle) (see Supplementary Fig. 8a for further details). We note that the low PCN of ancestral pCON plasmids at 37 °C resulted in lower plasmid yields and increased co-eluted chromosomal DNA (linear fragments, Supplementary Fig. 9a,c). **c** Genetic map of the plasmid pIND. **d** Plasmid topology of pIND plasmids (multimers and monomers, Supplementary Fig. 8b, for original see Supplementary Fig. 10) from two ancestral host populations at 37 °C (analyzed as in (**b**)). **e** Relative transcription per plasmid of pIND-encoded genes *araC* and *rep* in the two different pIND replicates pIND1 and pIND2 at 37 °C (*n* = 3). **a**, **e** The center value of the boxplots represents the median, the boxes denote the interquartile range, and the whiskers represent minimum and maximum values. Source data are provided as a Source Data file

Our results reveal that the plasmid copy number (PCN) as well as the transcription level of plasmid-encoded genes was higher at 20 °C in comparison to 37 °C (Fig. 3a and Supplementary Fig. 6). Analyzing the relative transcription per plasmid showed that the transcription of both genes, *rep* and *nptII*, was higher at 20 °C in comparison to 37 °C (Fig. 3a), also when accounting for differences in gene dosage due to differences in PCN. Furthermore, PCN and gene transcription level are positively weakly correlated for the ancestral plasmids at 20 °C (*r*_*s*_ = 0.407, *P* = 0.041, using Spearman correlation) but not at 37 °C (*r*_*s*_ = −0.165, *P* = 0.439, using Spearman correlation). The low relative transcription per plasmid, as well as the absence of a dosage effect at 37 °C, demonstrates that the transcription of *rep* and *nptII* was hampered in this temperature regime. The low transcription level of *rep* was likely associated with the low PCN at 37 °C (Supplementary Fig. 6), as well as the high frequency of segregational loss observed in the evolution experiment (Fig. 1a). We note that the differences in transcription level in the two temperatures were not translated into a difference in the effect of pCON on host fitness, which was similar in both temperatures (Supplementary Fig. 1). In addition, we measured the relative transcription and copy number of the stably inherited plasmids that emerged in the evolution experiment. Our results show that the transcription level of both plasmid genes, as well as the PCN, were not significantly different between the populations evolved at 20 °C and their ancestors (Fig. 3a; *P* = 0.9323 using Wilcoxon test). However, the transcription of *nptII* and *rep* of the stably inherited plasmids evolved at 37 °C was significantly higher compared to their ancestors (Fig. 3a; *P* = 7.396 × 10^−7^ using Wilcoxon test) and positively weakly correlated with the PCN (i.e., dosage effect, *r*_*s*_ = 0.3, *P* = 0.041, using Spearman correlation). This result demonstrates that plasmid DNA was replicated and accordingly transcribed (*nptII* and *rep*), thus showing that conflicts between transcription and replication were eliminated in the evolved plasmid variant.

To further investigate the implications of transcription and replication conflicts to the evolution of stable plasmid inheritance, we explored the topological state of the plasmid DNA. Plasmid interconversion, i.e., enzymatic reactions that determine DNA topology, is highly relevant for successful transcription and replication (e.g., refs. ^49,50^), as well as DNA diffusion and spatial distribution in the cell^51^. Furthermore, it is known that a change in the environmental temperature directly affects the DNA topology in the cell^52^. Here we visualized the plasmid topology using chloroquine gel electrophoresis. Chloroquine alters the relative mobility of circular DNA fragments during electrophoresis depending on the topology, the degree of superhelicity, and DNA mass (e.g., monomer or multimer). Our results show that the plasmid topology was different between the growth temperatures and largely different between ancestral (unstable) and evolved (stable) plasmids (Fig. 3b). The DNA topology of the ancestral plasmids from both temperatures was characterized by a similar distribution of topoisomers (Fig. 3b). We observed plasmid molecules that correspond to plasmid dimers, multimers as well as plasmid monomers (Supplementary Fig. 8a). Notably, supercoiled plasmid monomers were only visible in ancestral plasmid extracted from 20 °C (Fig. 3b). Furthermore, comparing the ancestral and evolved plasmids, we observed a stark enrichment in supercoiled monomers as well as a reduction of multimeric forms in the evolved populations. Notably, plasmids of the same genome have naturally different superhelical states in the cell (see ladder of topoisomers in Fig. 3b). The plasmid variants that evolved full stability at 37 °C clearly revealed the presence of plasmid monomers which were absent in the ancestor. The enrichment of monomeric forms is in accordance with the observed dosage effect in the evolved plasmids (Fig. 3a) as well as the stable inheritance over time (Fig. 1a). The segmental duplication in the evolved plasmids likely alleviates the torsional stress and reduces conflicts between transcription and replication, thus leading to the production of plasmid monomers (Fig. 3b). This indicates that transcription of the plasmid accessory gene and plasmid replication are tightly linked with the plasmid topological state. Our results so far show that the coordination of plasmid gene transcription and replication dynamics is an important determinant of plasmid evolution.

### Silencing *nptII* affects plasmid copy number and topological state

To further investigate the impact of transcription and replication conflicts on plasmid maintenance, we reduced the transcriptional load of pCON by silencing the transcription of *nptII*. For this purpose, we constructed pIND, in which *nptII* was placed under the control of the inducible p_BAD_ promoter (3.9 kb, Fig. 3c). We expected that this influences plasmid properties such as DNA topology, PCN, and plasmid gene transcription. The visualization of the pIND topology revealed a different distribution of plasmid conformations among replicates and in comparison to pCON. We observed two plasmid types of either plasmid multimers only or monomeric topoisomers only in various conformations (from here on termed pIND1 and pIND2 replicate populations, respectivly; Fig. 3d and Supplementary Fig. 8b). The presence of plasmid multimers is expected to be reflected in an increased PCN variability. Indeed, we observed that the PCN of the multimeric plasmids was highly heterogeneous, ranging between 10 and 300 copies per cell (*n* = 30). In contrast, the PCN of the monomeric plasmids was significantly lower and ranged between 4 and 7 copies per cell (*P* = 3.799 × 10^−8^, *n* = 30, using Wilcoxon test). Re-introduction of the two different pIND types into naive hosts showed that the PCN distribution remained similar, demonstrating that the plasmid form was a heritable trait (Supplementary Fig. 11a). To gain further insights into the source of pIND heterogeneity among replicates, we examined the genomes of the two populations. Differences in the coverage of the plasmid and chromosome validated our estimate for the PCN (Supplementary Table 3). Nonetheless, no differential genetic variants were observed in the host chromosome or the plasmid genome.

To test for differences in the transcription level between the two plasmid replicate populations, we measured the transcription of the plasmid-encoded genes *araC* and *rep* (Fig. 3c; note that *nptII* transcription remained silent). Our results show that the high PCN of the plasmid multimers was not correlated with the *araC* and *rep* transcription level (*r*_*s*_ = 0.153, *P* = 0.7713, *n* = 6, using Spearman correlation), whereas for the plasmid monomers the gene transcription level was significantly positively correlated with the copy number (*r*_*s*_ = 0.901, *P* = 0.01411, *n* = 6, using Spearman correlation). Accordingly, the transcription level per plasmid was higher for the monomeric plasmids in comparison to the multimeric plasmids (Fig. 3e). The lack of gene dosage effect in the multimeric plasmids demonstrates that transcription is hampered in plasmids having that topological form. Notably, the induction of *nptII* transcription with L-arabinose led to a decrease in PCN regardless of the plasmid topological state (*P* = 4.19 × 10^−12^, using Wilcoxon test, *n* = 3 isolates per replicated population, Supplementary Fig. 11b). This demonstrates that transcription of the plasmid-encoded resistance gene interferes with plasmid replication. Our results thus show that the elimination of the plasmid accessory gene transcription led to an alteration in the plasmid properties.

### pIND persistence is determined by plasmid resolution

The molecular properties of pIND suggest that transcriptional load has an impact on the long-term plasmid persistence. To investigate the long-term evolutionary dynamics of pIND we conducted an evolution experiment under non-selective conditions as described above for pCON. The effect of pIND on host fitness was evaluated by competition experiments and revealed that pIND had no measurable fitness cost in either of the temperature regimes (37 °C: *w* = 1.126, H_0_:*w* ≥ 1, *P* = 1.766 × 10^−12^ using Wilcoxon test, *n* = 64; 20 °C: *w* = 1.012, H_0_:*w* ≥ 1, *P* = 0.01471 using Wilcoxon test, *n* = 32; Supplementary Fig. 12).

**Fig. 4:**
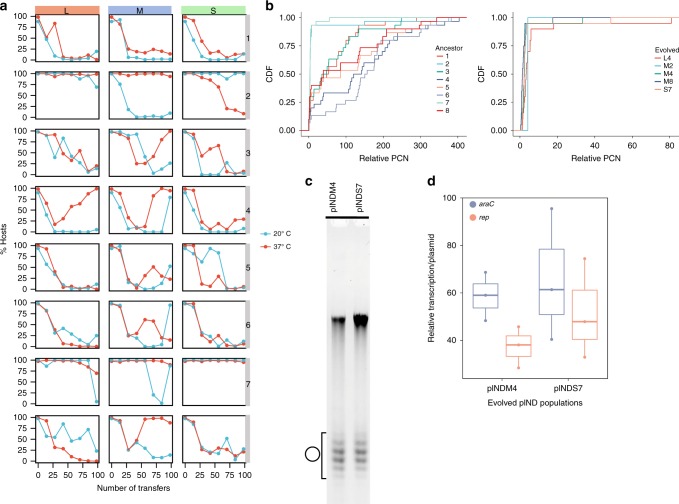
Evolution of pIND under non-selective conditions. Note that the expression of the plasmid encoded *nptII* gene was not induced during the evolution experiment. **a** pIND persistence is shown as the proportion of hosts during the evolution experiment at 20 °C (blue) and at 37 °C (red). The columns correspond to the three population bottleneck sizes (L, M, and S). The experiment was conducted for 98 transfers, which correspond to 600 generations in L populations, 800 generations in M populations, and about 1000 generations in S populations. Eight replicate lines are presented in vertical order (i.e., populations in each row have a common ancestral population). Replicates pIND1 and pIND2 correspond to the replicates presented in Fig. 3. **b** Cumulative distribution function of relative plasmid copy number (PCN) distribution in pIND ancestral populations (left) and evolved stable populations (right). Replicates correspond to the replicates in the evolution experiment in (**a**) (*n* = 30 or *n* = 20 for each population). **c** Evolved stable pIND plasmids from populations pINDM4 and pINDS7 (37 °C) analyzed by one-dimensional chloroquine gel electrophoresis (as shown in Fig. 3b, c). Symbol denotes the supercoiled monomer distribution of pIND (Supplementary Figs. 8b and 9b). **d** Relative transcription per plasmid of pIND from evolved populations pINDM4 and pINDS7 (37 °C, *n* = 3). The center value of the boxplots represents the median, the boxes denote the interquartile range, and the whiskers represent minimum and maximum values. Source data are provided as a Source Data file

The results of the evolution experiment revealed that pIND host frequency dynamics were heterogeneous among the replicates across different population sizes and growth temperatures (Fig. 4a and Supplementary Fig. 13). We observed frequent plasmid loss, yet the plasmid host frequency dynamics were stochastic, where neither temperature (Supplementary Fig. 13a), nor population size (Supplementary Fig. 13b) appeared to have a strong effect on plasmid loss. The proportion of host frequency in populations of the plasmid multimers, pIND1 (replicate population in Fig. 3d) decreased monotonically, while in populations of the plasmid monomers, pIND2 (replicate population in Fig. 3d), the plasmid remained overall stable (Fig. 4a). In addition, plasmid persistence was observed in population pIND7, where the host frequency remained high throughout the experiment. Further quantification of the plasmid stability of the persistent host populations showed that the frequency of plasmid-free cells after overnight growth was 0.5 ± 0.3% (SE, *n* = 3) for the ancestral pIND2 and no plasmid-free cells for the ancestral pIND7 population (*n* = 3). Notably, in several populations the decreasing plasmid frequency was reversed to an increasing trend with the plasmid hosts taking over the population (e.g., pINDM4). The increase in host frequency in these populations is likely explained by the increase in plasmid stability over time. Indeed, a quantification of the evolved pINDM4 loss frequency showed that only 0.2 ± 0.4% (SE, *n* = 3) plasmid-free cells appeared after overnight growth.

The multimeric form of pIND is characterized by a high and variable copy number (up to 400 copies per cell) while the plasmid monomers have a homogeneous low copy number (up to 7 copies per cell). A quantification of the PCN in all ancestral populations showed that plasmids in populations in which we observed a decreasing host frequency trend had a highly variable copy number while the stable populations had a low copy number (Fig. 4b). Furthermore, in the evolved population, in which the host frequency was stable, or increased with time, the PCN was comparably low (Fig. 4b). This result is in line with our observation on the relation between plasmid stability and the plasmid DNA form (Fig. 3). Indeed, the topological state of the evolved stable plasmids (pINDM4 and pINDS7) was similar among the replicates comprising mainly plasmid monomers (Fig. 4c and Supplementary Fig. 8b). Furthermore, the transcription level of the evolved stable plasmids was similar to the ancestral plasmid monomers (*P* = 4.955 × 10^−5^ using Wilcoxon test; Fig. 4d). Overall, the evolved stable pIND plasmids, including the plasmids that increased in frequency, were characterized by similar properties of copy number, transcription level and DNA topology.

We conclude that silencing of the accessory gene transcription eliminates the conflicts between transcription and replication and leads to reduced plasmid loss. Nonetheless, several pIND replicate populations encounter plasmid loss over time, hence, the plasmid conformation (i.e., multimer formation) remains an important determinant of plasmid heritability due to its implications for the unit of segregation. In a population that harbors heterogeneous plasmid forms, cells that are enriched for multimeric plasmids tend to have a higher plasmid loss rate due to uneven segregation^19^. Our results show that the monomeric form is an advantageous plasmid trait as it is crucial for stable plasmid inheritance and that natural selection acts in favor of the plasmid monomers. Such plasmids can be fixed in the bacterial lineage in the absence of selection for the plasmid-encoded trait.

## Discussion

Our study demonstrates that plasmid stability can evolve under non-selective conditions where plasmid intrinsic properties are important determinants of plasmid evolution. Plasmid gene transcription dynamics have been so far studied for their impact on the host physiology^53–55^. Focusing on the plasmid, our results show that plasmid gene transcription has implications for plasmid stability as accessory gene transcription may hinder plasmid replication and consequently also plasmid inheritance. Studies of the coordination between transcription and replication in bacteria showed that collisions of the replication and transcription machineries can lead to replication fork stalling, premature transcription termination and DNA double strand breaks that disrupt the genome integrity^56^. The competition between transcription and replication for the same DNA template leads to increased mutagenesis and reduced genome integrity due to duplication and deletions events^57,58^. Indeed, the segmental duplication that led to plasmid stability in our evolution experiment occurred in a genomic location that is a potential hotspot for transcription–replication conflicts. We hypothesize that small plasmids frequently encounter transcription–replication conflicts (i.e., torsional stress) following the acquisition of new genes due to their condensed genome architecture. For example, the insertion of mobile genetic elements such as transposons and IS elements that are frequently observed in plasmids (reviewed in ref. ^59^) may be such a case, where their gene transcription can disrupt plasmid replication and thus lead to plasmid instability and extinction. Furthermore, transcription–replication conflicts may have implications for the DNA template interconversion and the DNA conformation. Indeed, we observed different plasmid topoisomers in the presence or absence of constitutive transcription. The plasmid molecule form has consequences for the plasmid diffusion rate^51,60^ and hence for the plasmid spatial distribution within the host cell. Furthermore, previous studies have recognized the formation of plasmid multimers as an important determinant of plasmid instability^19,61^. Plasmids encoded multimer-resolution systems were shown to increase plasmid persistence over time (e.g., a resolvase encoded on a transposon^62^). Notably, the plasmid conformation in our experiment is not a genetic trait, yet we find that it is heritable characteristic and an important determinant of plasmid inheritance. Thus, intrinsic plasmid architecture and gene transcription dynamics play a role in plasmid evolution due the impact on the plasmid topology and the inherent constraints on the acquisition and distribution of protein coding genes in the plasmid genome.

The intrinsic properties of isogenic plasmids may change in different temperatures, which can lead to different plasmid evolution dynamics depending on the environment. Our results reveal that different temperature regimes have an effect not only on the host physiology but also on the plasmid maintenance where cold temperature appears as favorable for plasmid persistence. We hypothesize that cold temperature may have an effect on plasmid transcription and replication efficiency due to different factors including, for example, altered DNA topology, overexpression of the stress-induced repair mechanisms (which may be important for replication restart^63,64^), or decreased growth rate (i.e., metabolism). A combination of those factors may reduce the potential for conflicts of plasmid transcription and replication and hence lead to stable plasmid replication and inheritance. Our study demonstrates that conditions which are unfavorable for the host may be favorable for the plasmid, hence environmental conditions should be considered as another potential determinant of plasmid persistence.

Fluctuations in selective conditions for the plasmid maintenance can lead to increased plasmid persistence over time^26,32^. Our results show that plasmids have the potential to persist in a population in the absence of selection at a small frequency for long time scales (Fig. 2). Imposing selection pressure for the plasmid presence in such mixed population of hosts and non-hosts leads consequently to a strong population bottleneck for plasmid hosts only. Thus, a selective event constitutes a selective sweep of alleles that are represented in the plasmid host population, whereas chromosomal alleles of non-hosts are purged from the population. Such selective sweeps are expected to lead to rapid adaptation of the host and the plasmid, and consequently plasmid fixation in the population. We propose that this scenario of plasmid-driven evolution is frequent in natural environments where fluctuating conditions play a role in increasing the frequency of rare chromosomal variants in favor of the plasmid maintenance. The interplay between plasmid stability and host genetic diversity may well explain the increase in plasmid stability observed in experimental evolution of plasmids under fluctuating selection regimes^26,32^.

**Fig. 5:**
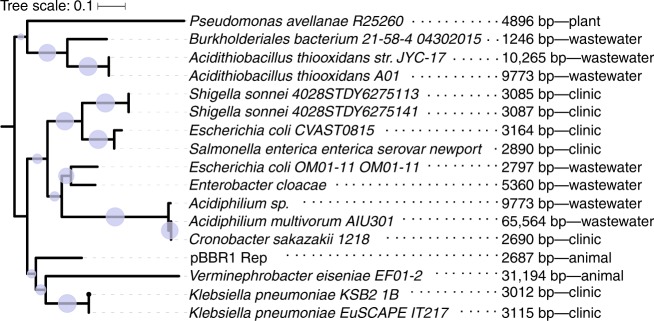
Phylogeny of pBBR1 Rep protein. A search for homologs of pBBR1 revealed several plasmids having a homologous Rep protein (≥65% identical amino acids). Phylogenetic relations between the Rep homologs are depicted by the tree topology with blue circles whose size is proportional to the bootstrap support in each node. Plasmid size and isolation location are indicated next to the host taxonomic name

The plasmid backbone in our experiment, pBBR1, was isolated from a virulent *Bordetella bronchiseptica* strain and is considered a broad host-range plasmid^39^. To test for the prevalence of similar plasmids in the environment we searched for plasmids whose replication initiation protein (Rep) is homologous to the pBBR1 Rep. This revealed several closely related plasmids found mainly within Gammaproteobacteria, several of the plasmids were isolated in clinical studies of nosocomial infections by antibiotic-resistant bacteria (Fig. 5 and Supplementary Table 4). The pBBR1 backbone is thus found in nature and it may play a role in the dissemination of antibiotic resistance. Notably, the majority of plasmids encoding pBBR1 Rep homologs is small, including plasmids that have a condensed genome architecture, similarly to the plasmids in our experiment. Thus, the evolutionary dynamics of pCON and pIND plasmids—even if artificially created—demonstrate a plausible scenario for plasmid evolution in a natural setting.

Plasmids are often considered as costly to their host fitness and are therefore expected to be eliminated from the lineage by purifying selection. We demonstrate that a small plasmid can be stable in its host regardless of the PCN or plasmid gene transcription dynamics. The plasmid effect on the host fitness (i.e., selection coefficient) often depends on the environmental conditions, which can act against—or in favor of—the plasmid maintenance. Notwithstanding, plasmids can persist in the population depending only on the efficiency of their inheritance (i.e., stability) and may reach fixation due to genetic drift, similarly to neutral alleles. Our study shows that the evolution of plasmid stability under non-selective conditions maintains antibiotic resistance of the host and offers an alternative explanation to the plasmid paradox and the high abundance of plasmids in nature.

## Methods

### Bacterial strains, plasmids, and culture conditions

The strain *E. coli* K-12 MG1655 was used as the model organism in all experiments (DSM No. 18039, German Collection of Microorganisms and Cell Cultures, DSMZ). For the purpose of competition experiments, *E. coli* MG1655 was equipped with a chromosomal mini-Tn*7* insertion (attTn7::miniTn*7(dhfrII)*) using a transposon vector developed by^65^ conferring resistance to trimethoprim (Tm^r^). The strain *E. coli* DH5α^66^ was used during plasmid construction. All strains were routinely grown at 37 °C in Luria Bertani (LB) medium at 250 rpm shaking. For molecular cloning and documentation purpose, the plasmid carrying strains were grown either in LB supplemented with 25 µg kanamycin per ml (pCON hosts) or supplemented with 5 µg kanamycin per ml and 1% L-arabinose (pIND hosts).

All plasmids in this study were constructed using the NEBuilder® protocol (New England Biolabs). The model plasmids pCON and pIND are comprised of the pBBR1 backbone (*rep* and *oriV*) that was amplified from the plasmid pLC (GenBank accession no. MH238456)^67^ using the primer pair pBBR1_for/rev (for primer see Supplementary Table 5). Both plasmids were equipped with the *nptII* gene encoding for a neomycin phosphotransferase and conferring resistance to kanamycin. For pCON, the *nptII* gene including its native *Tn5* promoter^40^ was amplified using the primer pair nptII_gib_for/rev. The plasmid was assembled containing the pBBR1 backbone and the *nptII* resistance cassette (2.6 kb; GenBank accession no. MK697350). For the construction of the plasmid pIND, the *nptII* gene (primer: nptII_for/rev) was sub-cloned into the vector pBAD30^68^ that contains the *ara*-operon using restriction enzyme cloning (cutsites: EcoRI and HindIII). Thereafter, the *ara*-operon containing the *nptII* gene was amplified from pBAD30 using the primer pair pBAD_gib_for/rev. Subsequently, the pBBR1 backbone and the *ara*-*nptII*-operon were assembled (3.9 kb; GenBank accession no. MK697351). All plasmids were extracted using the GeneJET Plasmid Miniprep Kit (Thermo Fisher Scientific) and DNA concentrations were measured using a NanoDrop^TM^ (Thermo Fisher Scientific).

For the validation of the stability of the evolved plasmid variant, we constructed the plasmids pCON-dup and pCON-ran. For this purpose, we conducted inverse PCR of pCON to amplify the plasmid (nptII_rev and pBBR1_for). In addition, we amplified either the segmental duplication (pCON-var_gib_for/rev) or a random DNA fragment from the pBBR1 *mob* pseudogene of pBBR1-MCS5^69^ of 500 bp (pCON-ran_gib_for/rev). The fragments were assembled using the NEBuilder protocol.

### Fitness experiments

The relative fitness (*w*)^70^ of the plasmid-carrying versus the ancestral plasmid-free strain (wt) was estimated by direct pairwise competition experiments, with eight replicate strains per plasmid type and temperature. The impact of the marked *E. coli* MG1655 Tm^r^ strain was evaluated by competition experiments of the wildtype against the marked strain (*n* = 8,  at 37 and 20 °C). All competition experiments were initiated with a 1:1 mixture of 1:100 diluted plasmid-carrying strain and ancestral strain (Tm^r^) from overnight cultures, in a total volume of 1 ml of non-selective LB medium. The relative fitness of the plasmid host strains was calculated by gaining viable cell counts at the time points 0 and 24 h. The strains were distinguished through plating on non-selective (LB) and selective media (LB supplemented with 150 µg trimethoprim per ml and LB supplemented with 25 µg kanamycin per ml (pCON-carrying) or 5 µg per ml and 1% L-arabinose (pIND-carrying)). The plating for two marker genes (chromosomal and plasmid) enabled a control for potential plating errors and detection of plating inefficiency. For each of the eight populations the experiment was carried out with eight (37 °C, *n* = 64) or four (20 °C, *n* = 32) technical replicates (Supplementary Figs. 1 and 12).

### Evolution experiment

The evolution experiment was conducted with plasmid-carrying strains under non-selective conditions in two temperatures and three population bottleneck sizes. On the onset of the experiment, the pCON and pIND host strains were plated on selective media to ensure plasmid carriage. The experiment was founded by eight replicate colonies of each plasmid type which were inoculated in LB medium at 37 °C constant shaking. After overnight growth all cultures were diluted 1:100 (large (L) bottleneck), 1:1000 (medium (M) bottleneck), or 1:10,000 (small (S) bottleneck) and transferred into two 96-deep-well plates in a total volume of 1 ml. The diluted cultures were either incubated at 37 or 20 °C with constant shaking. To account for the effect of growth temperature on the growth dynamics, populations cultured at 37 °C were transferred every 12 h while those cultured at 20 °C were transferred every 24 h. During every transfer event the bottleneck size treatment was applied and the serial transfer was repeated over a total of 98 transfers. Note that the serial population bottleneck determined the total number of generations of the populations. The number of generations was routinely measured by the evaluation of the cell number through plating directly after the dilution and before the next subsequent transfer. We observed a total number of ~600 generations (6 generations per transfer) for the large population, ~800 (8 generations per transfer) for the medium population and ~1000 (10 generations per transfer) for the small population. The number of generations was similar for both temperature regimes as the time between transfer events was adjusted accordingly. During the evolution experiment, the frequency of plasmids in the population was estimated from the proportion of hosts, which was determined by replica plating as described by^71^ every 14 transfers. Briefly, this was performed by first serially diluting the grown cultures followed by plating of ~500 cells on non-selective LB media. The number of plated cells was increased with decreasing plasmid frequency up to ~1000 cells. The plated populations were incubated for overnight growth according to their growth temperature in the experiment. Colonies were counted and replicated using velvet cloth on selective media according to their plasmid type (LB supplemented with 25 µg kanamycin per ml for pCON host cultures and LB supplemented with 5 µg kanamycin per ml and 1% L-arabinose for pIND hosts cultures). Colonies growing on the selective media were counted as plasmid hosts.

To test for pCON stability evolution, the evolved populations from the 37 °C pCON experiment were transferred into a 96-well-plate containing selective media (LB supplemented with 25 µg kanamycin per ml) and incubated for overnight growth (12 h). Thereafter, the cultures were transferred into non-selective conditions for a follow-up evolution experiment at 37 °C along a total of 56 transfers. Plasmid host frequency was monitored via replica plating.

### Single-colony transfer experiment

Single-colony transfer of pCON hosts was performed at 37 and 20 °C. The experiment was conducted under non-selective conditions on LB-agar plates. On the onset of the experiment, the pCON hosts were plated on selective media to ensure plasmid carriage and the experiment was started with 30 randomly chosen colonies. These replicate colonies were streaked on non-selective media. In every transfer, a randomly picked colony of each replicate was transferred to a fresh plate. To monitor plasmid abundance each streaked colony was in parallel transferred to a selective plate and only further transferred if the plasmid was present; otherwise the number of host was reduced. To account for different growth dynamics in the different temperatures, the transfer was conducted every 24 h for colonies cultured at 37 °C and every 48 h for colonies cultured at 20 °C. The experiment was conducted for a total of 33 transfers. The number of generations was estimated by suspending and plating the streaked colonies followed by evaluating the cell numbers. This resulted in approximately 800 generations (ca. 24 generations per transfer).

### Plasmid loss frequency assays

The plasmid loss frequency was estimated from the frequency of plasmid-free cells occurring during overnight growth in non-selective media. To determine the plasmid loss frequency, cultures were inoculated from single colonies grown on selective media to ensure plasmid carriage. After 12 h growth in 37 °C or 24 h in 20 °C (approximately 8.5 generations), the cultures were serially diluted and plated on non-selective media. After overnight incubation the plates were replicated using the protocol by ref. ^71^. Briefly, colonies from non-selective plates were transferred to a velvet cloth and replicated onto a selective plate. After overnight growth colonies were counted as plasmid carrying. This was validated by manually picking 100 colonies from non-selective to selective media. The loss frequency was calculated from plasmid-free cells (not resistant) and the total number of colonies tested.

### Plasmid copy number determination

The PCN was determined using quantitative real time PCR (qPCR) as described in ref. ^72^. Briefly, the bacterial cells were lysed by 10 min incubation at 98 °C followed by 10 min at −20 °C. The qPCR was conducted with primers targeting the chromosome and the plasmid. The chromosomal primers were complementary to *idnT* of *E. coli* (q_idnT_F/R)^73^ and the plasmid primers targeted the *nptII* gene (q_nptII_F/R). The qPCR reactions were conducted in volume of 10 µl containing 1× iTaq Universal SYBR Green Supermix (Bio-Rad Laboratories), 100 nM of each primer (final concentration) and 1 µl sample. All qPCR reactions including positive and non-template controls were performed in technical replicates on a CFX Connect™ Real-Time PCR Detection System (Bio-Rad Laboratories) using the following cycling conditions for all reactions: 95 °C for 3 min, and 40 cycles of 10 s at 95 °C and 1 min at 59 °C. Primer specificity and efficiency were determined using standard-curve and melt-curve analyses^72^. The ratio between the number of plasmid amplicons and chromosome amplicons is defined as the PCN (comparative C_T_ (ΔΔC_T_) method) and was here calculated while considering the amplification efficiencies of both primer pairs.

### Quantification of gene transcription level

The total RNA of the plasmid-carrying strains was isolated using the Direct-zol RNA MiniPrep kit (Zymo Research). Briefly, the populations were plated on selective media to isolate singles colonies for overnight growth. The cultures were either incubated at 37 °C for about 12 h growth or at 20 °C for about 24 h growth. The cells were harvested at a similar optical density (OD_600_) in the late exponential phase. Subsequently, RNA extraction of 1 ml cell culture was performed according to the provided protocol. To eliminate genomic DNA contamination, an on-column DNase I digest (Zymo Research) was performed during the RNA isolation procedure. The RNA concentration was measured using the NanoDrop^TM^ (Thermo Fisher Scientific) and RNA integrity was validated by gel electrophoresis. Afterwards, an additional DNase treatment using the Ambion™ DNase I kit was performed, according to the manufacturer instructions. To ensure complete plasmid DNA removal, the RNA was incubated for a double DNase digestion (2 units) at 37 °C for 1 h. For reverse transcription, cDNA samples were synthesized from 500 ng of total RNA templates using the qScript cDNA Synthesis Kit (Qantabio, USA) following the manufacturers protocol. Negative control reactions containing all components for reverse transcription with the exception of the reverse transcriptase enzyme, were performed to verify the absence of chromosomal or plasmid DNA in the RNA samples. To quantify the relative transcription of the plasmid genes, reverse transcription quantitative PCR (RT-qPCR) targeting the plasmid genes and the chromosomal marker *idnT* was performed. The RT-qPCR assays were performed using the primers and conditions as described for the plasmid copy determination. The relative transcription level was determined using the comparative C_T_ (ΔΔC_T_) method where plasmid gene transcription is calculated relative to the house-keeping gene (*idnT*) and per plasmid in the cell (i.e., dividing by the PCN).

### One-dimensional chloroquine gel electrophoresis

The electrophoretic mobility of circular plasmid molecules depends on their mass (monomeric or multimeric forms of pCON and pIND), topological state defined by the linking number of number of the complementary strands and knot type, and the degree of catenation. We used 1-dimensional gel electrophoresis in the presence of chloroquine to resolve different topological forms of supercoiled plasmids. For one-dimensional gel electrophoresis, plasmid DNA preparations, obtained from stationary cultures, were electrophoresed at 5 °C in 1% (w/v) agarose gels at 1.9 V/cm in 1× TBE buffer containing 4 µg per ml chloroquine for 20–30 h with continuous buffer recirculation. Chloroquine was added to the molten agarose at the same concentrations as in the electrophoresis buffer prior to casting the gel. Chloroquine is a planar molecule that can intercalate into the DNA double helix. In covalently closed DNA molecules, the intercalation causes a reduction in helical twist (unwinds the DNA) and an increase of DNA writhe through compensatory positive supercoils. As a result, positively supercoiled molecules are further compacted, while negative molecules become relaxed. At lower concentrations of chloroquine, as used here, the increase in writhe does not remove all negative supercoils in negatively supercoiled plasmids, but leads to a compaction of these molecule species. As in *E. coli* in vivo plasmids are almost always negatively supercoiled, faster migrating topoisomers likely reflect negatively supercoiled molecules. All gels were destained by soaking in distilled water (3 × 1 h at 5 °C) to remove chloroquine, stained with ethidium bromide (1 µg per ml; 1 h at 5 °C) and finally destained with 1 mM MgSO_4_ for (2 × 1 h at 5 °C) before imaging on a ChemiDoc Gel Imaging System (Bio-Rad Laboratories). Original images for all gels are shown in Supplementary Figs. 7, 8, 9d, and 10.

### Genome sequencing

Population sequencing was used to detect genetic variants occurring either on the plasmid or the host chromosomes. Plasmid DNA was extracted from 3 ml stationary culture using the GeneJET Plasmid Miniprep Kit (Thermo Fisher Scientific). Total DNA was isolated from 1 ml culture using the Wizard Genomic DNA Purification Kit (Promega). Concentration and quality of the extracted plasmid and genomic DNA was assessed using the NanoDrop^TM^ (Thermo Fisher Scientific) and Qubit (Invitrogen by Life Technologies). The sample libraries for Illumina sequencing were prepared using the Nextera XT library kit (Illumina, Inc.) and sequencing was performed with paired-end reads on the Miseq system (Illumina, Inc.).

Sequencing reads were trimmed to remove Illumina specific adaptors and low quality bases using the program Trimmomatic v.0.35^74^ (parameters: ILLUMINACLIP:NexteraPE-PE.fa:2:30:10 CROP:250 HEADCROP:5 LEADING:20 TRAILING:20 SLIDINGWINDOW:4:20 MINLEN:36). As the reference we joined the *E. coli* MG1655 (GenBank accession no. NC_000913.3) genome with the pIND plasmid sequence (created in SnapGene software v.2.4 (GLS Biotech)). For the pCON plasmids we used the validated pCON sequence as a reference. The sequencing reads were mapped to the reference genomes using BWA-MEM v.0.7.5a-r405^75^. Mapping statistics were retrieved using BAMStats v.1.25 (https://sourceforge.net/projects/bamstats/files/). Subsequent indexing, local realignment of sequencing reads were performed using PICARD tools, SAMtools v.0.1.19^76,77^ and GATK v.3.6^77^ retaining only paired mapped reads with a minimum mapping quality of 20. SNPs were called using LoFreq v.2.1.2^78^ and GATK^77^. PCN was inferred from the de BAM files as the ratio of plasmid to chromosomal mean coverage. The coverage distribution was further used to infer large structural variants of the plasmids.

### Phylogenetic analysis

The search for homologs of pBBR1 Rep (RefSeq accession: YP_009062864.1) was performed with BLAST^79^ in two approaches: (1) using the stand-alone version (ver. 2.2.26) against the RefSeq of completely sequenced prokaryote genomes (ver. 06/2018), and (2) using the NCBI online interface against the RefSeq plasmids database (ver. 02/2019). From among the identified similar Rep sequences (Supplementary Table 4), protein sequences having ≥65% identical amino acids to pBBR1 Rep were aligned with MAFFT^80^ using the default parameters. The resulting multiple sequence alignment was used for the reconstruction of a maximum-likelihood phylogenetic tree using IQTree^81^ with LG model and 1000 bootstrap replicates. A rooted topology of the resulting phylogenetic tree was inferred with MAD^82^ (the root ambiguity index (AI) was 0.875). The phylogenetic tree was plotted using iTOL^83^.

### Statistical analysis

All statistical tests and data analysis were performed in R version 3.5.1.

### Reporting summary

Further information on research design is available in the Nature Research Reporting Summary linked to this article.

## Supplementary Information

Supplementary Information

Reporting Summary

Source Data

## Data Availability

All data in this study are available from the corresponding authors upon request. The source data underlying Figs. 1–4 and Supplementary Figs. 1–4, 6, 11–13 are provided as a Source Data file. Plasmids are available under GenBank accession no. MK697350 for pCON and MK697351 for pIND. All sequencing reads are available at the NCBI SRA database under the accession of PRJNA534303.
