# Supplementary material for: Emergence of plasmid stability under non-selective conditions maintains antibiotic resistance
Source: Nat Commun. 2019 Jun 13;10:2595. doi: 10.1038/s41467-019-10600-7 (PMC6565834; doi:10.1038/s41467-019-10600-7)
Supplement: Supplementary file 1 — Supplementary Information [file 41467_2019_10600_MOESM1_ESM.pdf]

Supplementary information

Emergence of plasmid stability under non-selective conditions maintains antibiotic resistance

Wein et al.

Supplementary figures

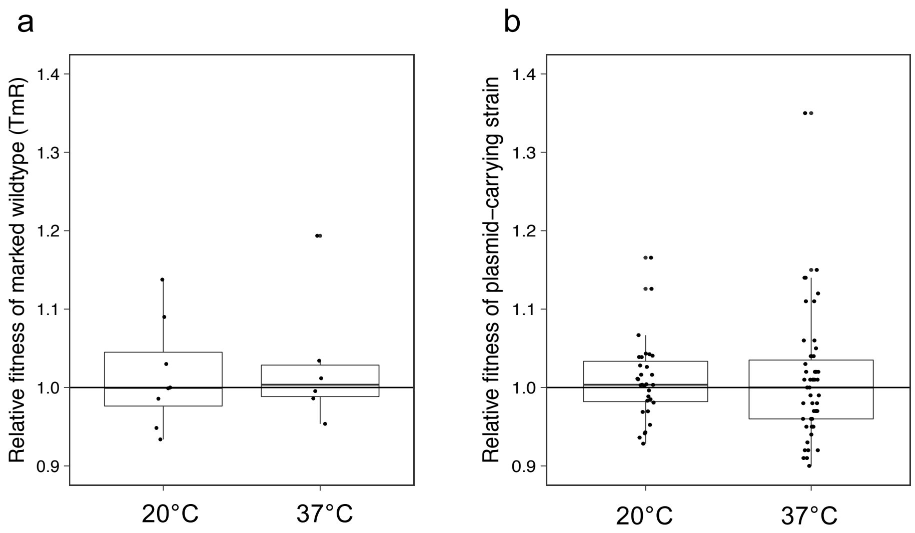

Supplementary Figure 1. Competition experiments to quantify the effect on the host fitness. **a**, Pairwise competition experiments between *E. coli* MG1655 wildtype and a chromosomally marked wildtype (Tm^r). **b**, Pairwise competition experiments between the plasmid-carrying (pCON) *E. coli* MG1655 and the marked wildtype (Tm^r). The experiments were conducted for 24h at 20°C and 37°C with eight biological replicates, including four technical replicates at 20°C (n=32) and eight replicates at 37°C (n=64). No significant fitness effect could be observed (20°C, *w*: 1.001, H_0:*w*≥1, *P*=0.009625 using Wilcoxon test; 37°C, *w*: 0.98, H_0:*w*≥1, *P*=0.0147 using Wilcoxon test). Moreover, the relative fitness was not significantly different between the temperatures (*P*=0.07699 using Wilcoxon test). The fitness experiments were performed measuring cell numbers for both marker types (wt: Tm^r and plasmid: Km^r). We did not observe a difference between the calculated (derived from chromosomal marker Tm^r) and measured plasmid marker Km^r (*P*=0.1914 using Wilcoxon-test). The center value of the boxplots represents the median, the boxes denote the interquartile range, and the whiskers represent minimum and maximum values. Source data are provided as a Source Data File.

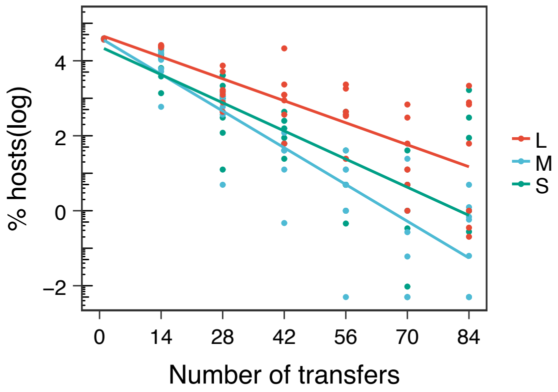

Supplementary Figure 2. Linear regression of pCON host dynamics at 37°C**.** Replicates of the evolution experiment were pooled according to the population bottleneck treatment and log transformed prior to fitting a linear regression.

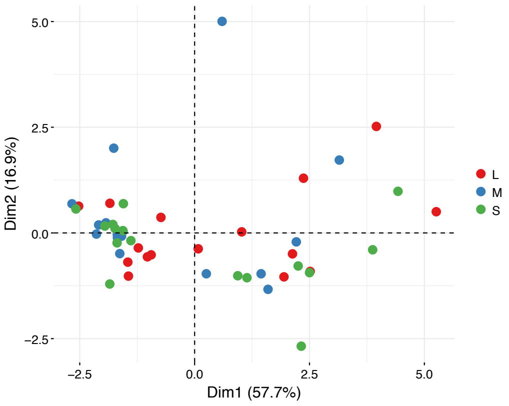

Supplementary Figure 3. Principal component analysis (PCA) of the pCON persistence over time comparing among population bottleneck sizes.

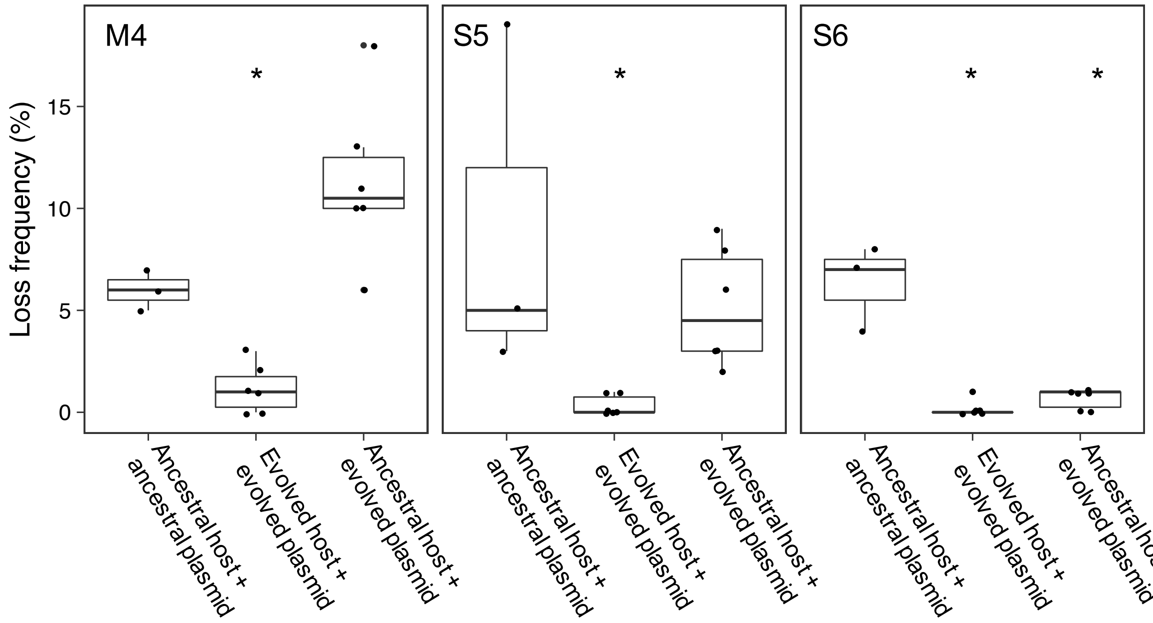

Supplementary Figure 4. Plasmid loss frequency of pCON populations in the follow-up evolution experiment. The plasmid loss was evaluated after overnight growth by measuring the number of plasmid segregants. Plasmid loss assays were conducted for the ancestral populations as well as for the evolved populations of population M4, S5 and S6. The evolved plasmid was introduced to the ancestral host and the loss was measured subsequently (n =3, n=6). The star indicates a significant difference in comparison to the ancestral loss frequency (<0.05 using Wilcoxon test). The center value of the boxplots represents the median, the boxes denote the interquartile range, and the whiskers represent minimum and maximum values. Source data are provided as a Source Data File.

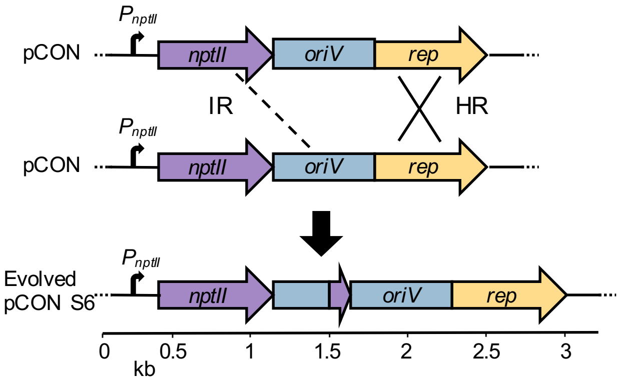

Supplementary Figure 5. Recombination event that led to the segmental duplication in pCON plasmids from population S6. The insertion was formed at a microhomologous region between *nptII* and the *oriV* through an illegitimate recombination event (IR, dashed line) that probably succeeded a distant homologous recombination event (HR) between two copies of pCON.

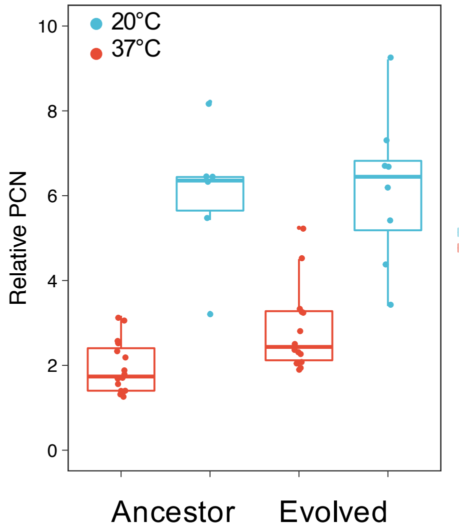

Supplementary Figure 6. Plasmid copy number of pCON plasmids. The plasmid copy number (PCN) was measured with quantitative PCR and calculated relative to the chromosomal gene *idnT*. The PCN for the evolved populations was evaluated from the stable population S6 at 37°C (n=16) and population L2 at 20°C (n=8). The center value of the boxplots represents the median, the boxes denote the interquartile range, and the whiskers represent minimum and maximum values. Source data are provided as a Source Data File.

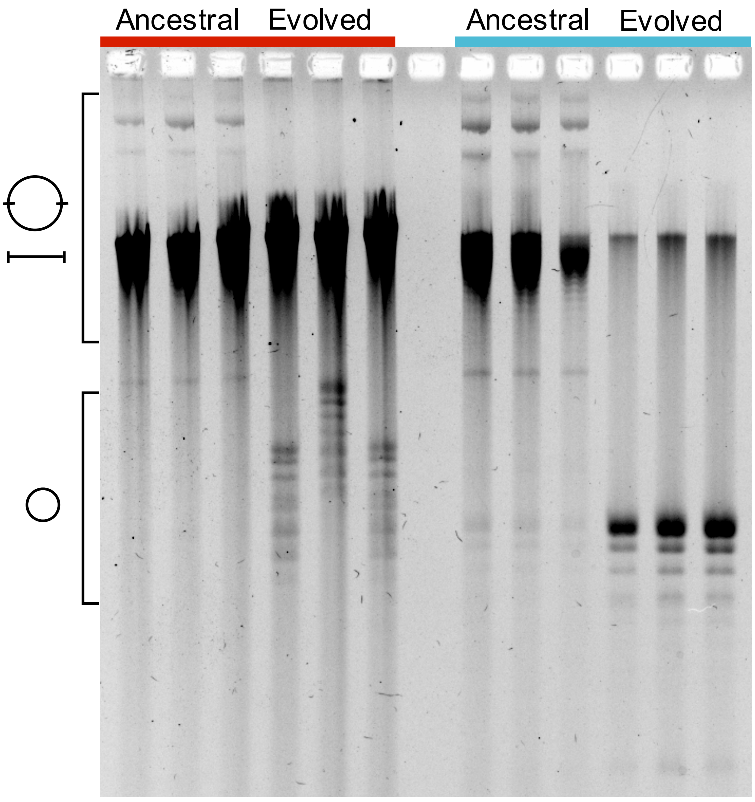

Supplementary Figure 7. Separation of pCON plasmid preparations by one-dimensional agarose gel electrophoresis in the presence of chloroquine (4 µg per ml). Plasmid preparations of ancestral and evolved populations grown at 37°C (red) and 20°C (blue). Symbols indicate plasmid topoisomers of multimers (large circle) and monomers (small circle) as well as linear DNA.

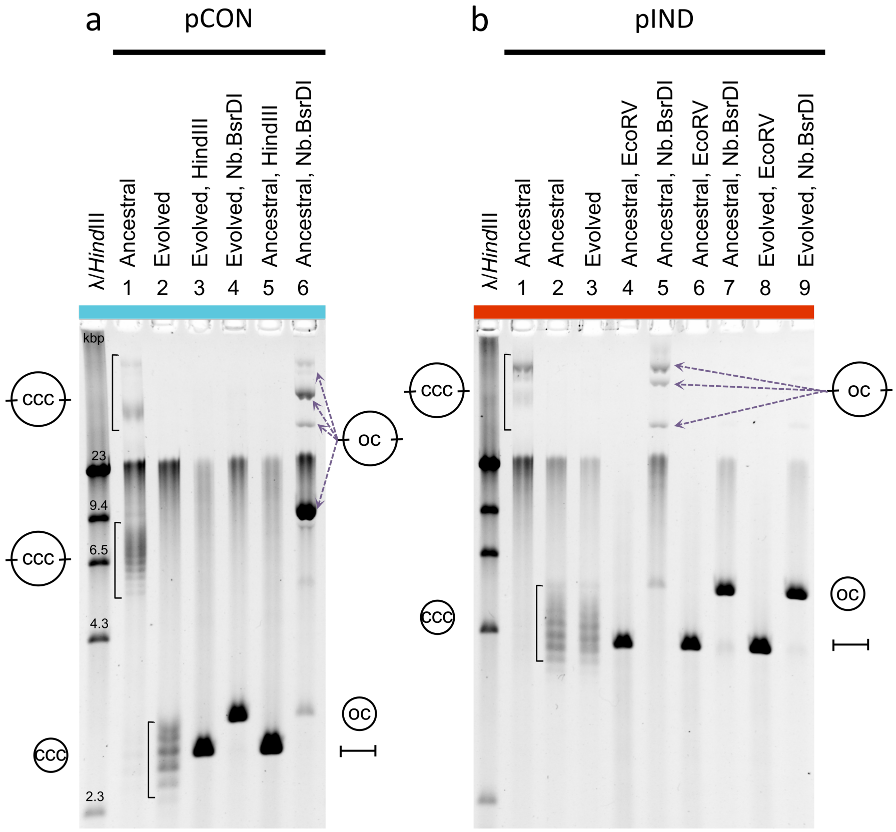

Supplementary Figure 8. Plasmid monomers and multimers identified by one-dimensional gel electrophoresis. Representative plasmid preparations of pCON and pIND were analysed by gel electrophoresis in the presence of choloroquine (4 µg per ml). The plasmids presented here were prepared from ancestral and evolved host lines from the 20°C (blue bar) and 37°C (red bar) regime. Plasmid DNAs were nicked with Nb.BsrDI to create relaxed open circular (oc) plasmid molecules. Linear monomer fragments were produced by digesting pCON with HindIII and pIND with EcoRV. Because chloroquine alters the relative mobility of circular DNA fragments during electrophoresis depending on the topology, linearized lambda DNA (λ) was included for a rough approximation but not as an accurate size standard. **a**, The vast differences in the relative mobility of the dominating fraction of topoisomers of pCON in the ancestral state (lane 1) and at the end of the evolution experiment (lane 2) are explainend by a dominating pCON multimer (lane 6) and not caused by different degrees of superhelicity. Note that the dominating oc form of the ancestral pCON multimer (lane 6) migrates close to the position of the first relaxed topoisomer of this multimer visible in lane 1. The treatment with HindIII converts all multimeric forms into linear molecules (lane 5). In addition to the dominating multimeric form, even larger multimeric forms of pCON exist *in vivo* in the ancestral lines at 20°C (i.e., slow migrating bands of oc multimer molecules (lane 6) that are not resolved when still supercoiled in lane 1). In contrast, pCON prepared from evolved lines appeared in a monomeric state (lane 3) as confirmed after nicking (lane 4) and linearization (lane 5). **b***,* Preparations of pIND from ancestral lines were either dominated by large plasmid multimers of different size (lane 1) or monomers (lane 2). Large multimers could not be resolved into topoisomers, but formed sharp bands after nicking (lane 5). Preparations of pIND from host lines after experimental evolution revealed uniformously that pIND was maintained *in vivo* in its monomeric state (lane 3). Nicking and linearization of pIND topoisomers from ancestral and evolved lines that maintained plasmids monomers revealed no differences (lanes 8 and 9).**Supplementary methods for Supplementary Figure 8:** Plasmids were isolated from overnight cultures grown without antibiotic selection at 20°C or 37°C using the GeneJET Plasmid Miniprep kit (Thermo Fisher Scientific). Enzymatic reactions were performed according to the manufacturers' guidelines (New England Biolabs). The samples were electrophoresed in a 0.7% (w/v) agarose gel for 700 to 900 min at 1.8 V/cm. Staining and image acquisition were performed as described in the main text.

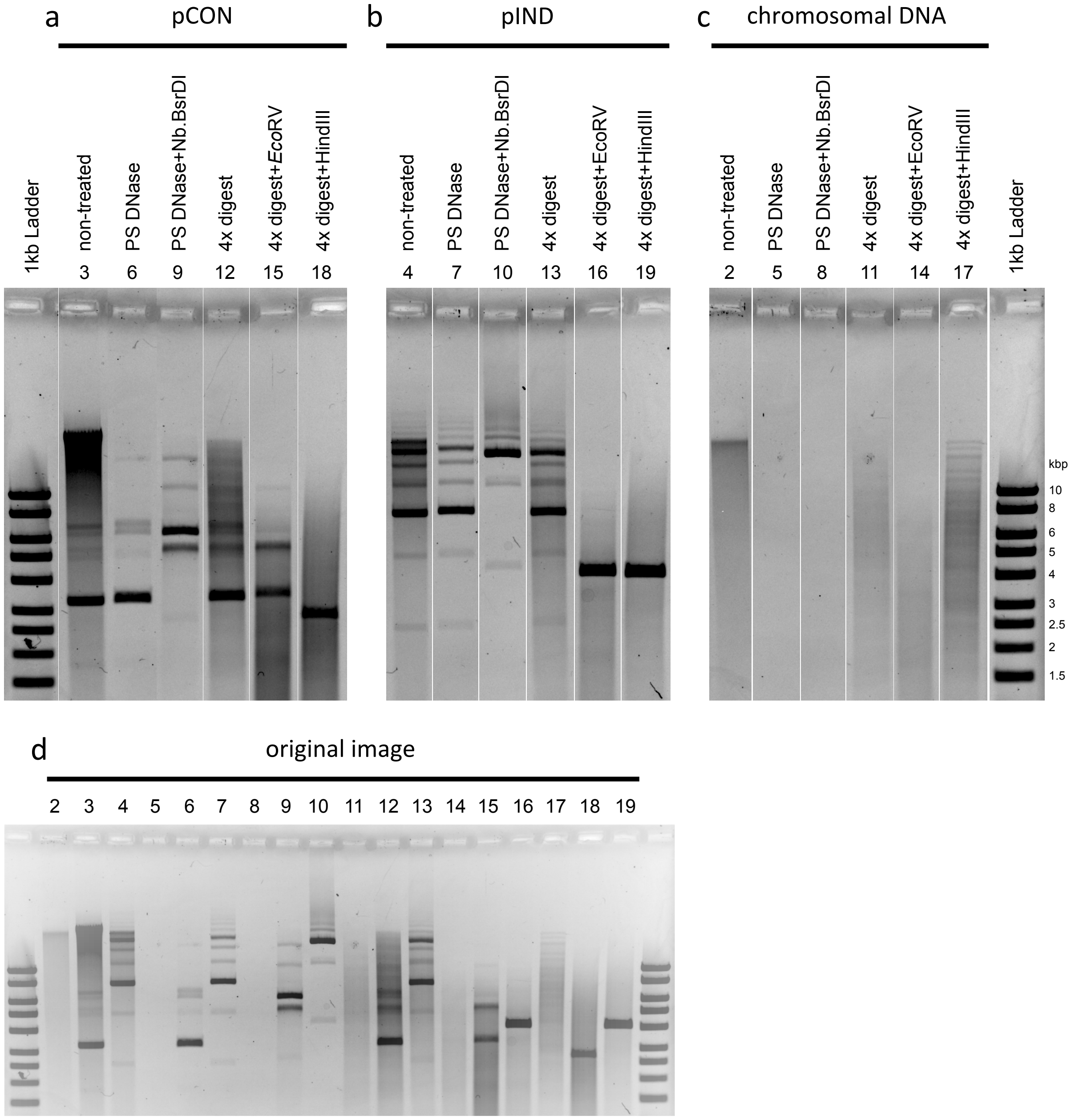

Supplementary Figure 9. Plasmid structures in ancestral plasmid DNA preparations of pCON and pIND. Representative plasmid preparations of pCON and pIND obtained from the ancestral *E. coli* lines (37°C regime) were assayed by native one-dimensional gel electrophoresis on a 0.7% agarose gel. Under this condition, supercoiled molecules (ccc), their nicked counterparts (oc) and linear fragments can be separated based on their size and mass. Treatment with Plasmid-Safe ATP-dependent DNase (PS-DNase) (Epicentre) was used to remove chromosomal DNA of the host from the samples. Open circular forms were obtained by nicking with Nb.BsrDI. Quadruple digests (4x digest) with AscI, NotI, NdeI, and XhoI (that do not cleave in pCON or pIND) were used to test for linear plasmid forms in the plasmid preparations. Treatments with EcoRV and HindIII in combination with the 4x digestion mix were used to produce linear plasmid monomers and to reduce the background signal of the chromosomal DNA in the samples. **a**, The high molecular weight DNA and DNA smear pattern obtained in a non-treated pCON plasmid preparation (lane 3) is comprised of linear double stranded DNA fragments (lane 6) of chromosomal origin (lane 12, 15, and 18).

Exonucleolytic removal of linear DNA (lane 3) reveals multimeric pCON molecules besides the dominating fraction of ccc pCON monomers. Nicked oc forms of pCON multimers confirm the presence of circular dimers and trimers of pCON (lane 9). **b***,* Multiple plasmid isoforms of pIND comprising different ccc and oc forms are readily observable in a non-treated preparation and after removal of linear fragments (lanes 4, 7, and 13) of chromosomal origin (lanes 13, 16, and 19). Different oc forms of pIND obtained after nicking (lane 10) confirm the presence of multimers. **c**, Control reactions performed with chromosomal DNA of *E. coli* MG1655. **d**, Original image. The lane numbering in **a**, **b**, and **c** refers to the lane numbering of the original gel.**Supplementary methods for Supplementary Figure 9.** Plasmids were isolated from overnight cultures grown without antibiotic selection using the GeneJET Plasmid Miniprep kit (Thermo Fisher Scientific). Genomic DNA was prepared using the Wizard genomic DNA purification kit (Promega). Enzymatic reactions were performed according to the manufacturers' guidelines (Epicentre, New England Biolabs). The samples were electrophoresed for 130 min at 4.3 V per cm. Staining and image acquisition were performed as described in the main text. The relative mobility of identified plasmid bands in each lane was mapped against the linear size standard (lane 1 and 20) to estimate their topological form (ccc, oc, or linear).

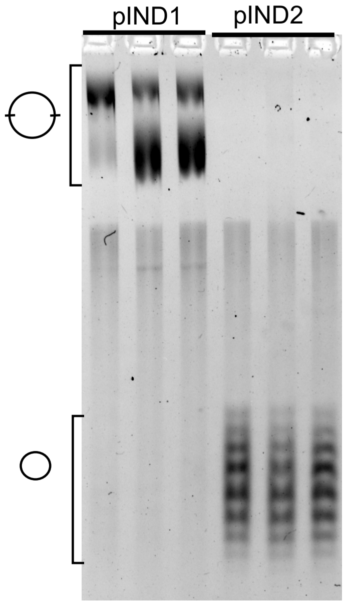

Supplementary Figure 10. Original chloroquine gel electrophoresis of pIND plasmid preparations of ancestral populations grown at 37°C. Symbols indicate plasmid topoisomers of multimers (large circle) and monomers (small circle).

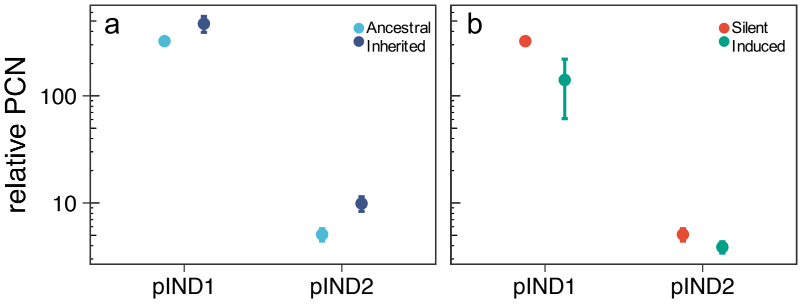

Supplementary Figure 11. The pIND copy number is heritable and is reduced upon induction plasmid gene transcription. The pIND replicates pIND1 and pIND2 correspond to pIND replicates in Fig. 3 and Fig. 4. **a**, Plasmids from pIND populations were re-introduced into naïve *E. coli* hosts. The relative plasmid copy number (PCN) was measured using qPCR (SE, n=3 per population) and is presented in log-scale. **b**, pIND plasmid copy number before and after induction of *nptII* transcription. The relative PCN of the pIND populations was measured after growth in LB (red, n=3, SE). Thereafter, the populations were transferred into LB supplemented with 1% L-arabinose and the PCN was measured subsequently (purple, n=3, SE). Result of the statistical comparison is reported in the main text. Source data are provided as a Source Data File.

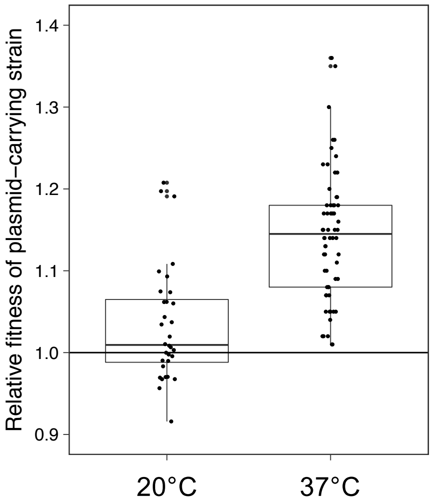

Supplementary Figure 12. Pairwise competition experiments between the plasmid-carrying (pIND) *E. coli* MG1655 and tagged wild-type (Tm^r). The experiments were conducted for 24 h in 20°C and in 37°C with eight biological replicates which were comprised of four technical replicates at 20°C (n=32) and eight replicates at 37°C (n=64). No significant fitness effect could be observed for the pIND plasmid (20°C: w=1.012, $H_0{:}w \geq 1$, P=0.01471 using Wilcoxon test; 37°C: w= 1.126, $H_0{:}w \geq 1$, P=1.766x10^{-12} using Wilcoxon test). The temperature causes a different fitness impact of pIND on its host (P=1.609x10^{-8} using Wilcoxon-test). We further tested the impact of the selective marker and did not detect an effect between calculated and measured values for pIND fitness (P=0.8919 using Wilcoxon-test). The center value of the boxplots represents the median, the boxes denote the interquartile range, and the whiskers represent minimum and maximum values. Source data are provided as a Source Data File.

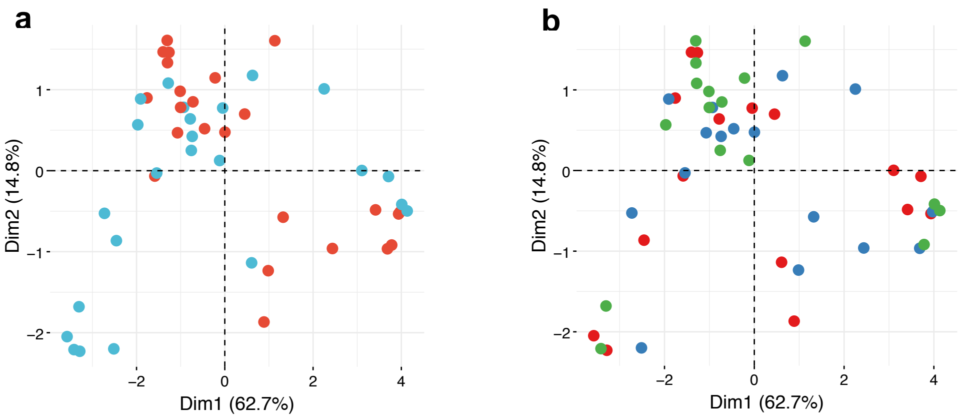

Supplementary Figure 13. Principal component analysis PCA of pIND host frequency over time. **a**, Effect of temperature on pIND host frequency over time. **b**, Effect of population bottleneck size on pIND host frequency over time.

Supplementary tables

Supplementary Table 1. Linear regression analysis of pCON growth dynamics from the evolution experiment (Fig. 1A) using log transformed data.

**Temperature**	**Population Bottleneck**	**Linear equation**	**y = 0**	R^2	**p-value**
37°C	L	y=4.69+(-0.04)x	117	0.8461	$2.2x10^{-16}$
	M	y=4.62+(-0.07)x	66	0.7996	$2.2x10^{-16}$
	S	y=4.36+(-0.053)x	83	0.6475	$3.252x10^{-12}$
20°C	L	y=4.52+(-0.019)x	237	0.2012	0.0005268
	M	y=4.18+(-0.019)x	220	0.1777	0.001499
	S	y=4.66+(-0.017)x	274	0.2786	4.881e-05

Supplementary Table 2. Data on genomic sequencing of evolved pCON plasmids.

**Population**	**Ref. size (Kb)**	**No. of reads**	**Mapped reads**	**Coverage Plasmid (±SD)**
pCON	2.601	362,005	28.5	6592±1652
pCONS6	2.601	351,520	7.43	1511±518

Supplementary Table 3. Data on genomic sequencing of ancestral pIND populations. No differential variants could be detected between the two populations.

**Population**	**Ref. size (Mb)**	**No. of reads**	**Mapped reads**	**Coverage Chromosome (±SD)**	**Coverage Plasmid (±SD)**	**Derived PCN**
pIND1	4.64	2,469,550	99.81%	68±17	5347±959	79
pIND2	4.64	1,914,564	99.95%	56±9	134±26	2.5

Supplementary Table 4. Phylogenetic analysis of pBBR1 Rep protein.

Strain	Strain isolation location	Plasmid genome sequence ID	Plasmid genome size (bp)	No. genes in plasmid genome	Rep homolog gene sequence ID	Similarity to pBBR1 Rep (%identical amino acids)
Klebsiella pneumoniae strain KSB2_1B	clinic	NZ_CP024505.1	3,012	2	WP_099743376.1	78
Klebsiella pneumoniae strain EuSCAPE_IT217	clinic	UKSJ01000049	3,115	3	SYD79493.1	78
Escherichia coli strain OM01-11	clinic	QSVV01000122	2,778	4	RGO66925.1	75
Shigella sonnei strain 4028STDY6275141	clinic	UIQG01000241	3,087	4	SVI62134.1	73
Shigella sonnei strain 4028STDY6275113	clinic	UDZO01000249	3,085	4	SRS26994.1	73
Burkholderiales bacterium 21-58-4 04302015	wastewater	NCAU01000378	1,246	1	OYV47792.1	71
Escherichia coli strain CVAST0815	clinic	NFTC01000081	3,164	3	RBU87737.1	75
Salmonella enterica subsp. enterica serovar Newport str.	clinic	JAMG01000017	2,890	3	OXX93233.1	75
Cronobacter sakazakii strain 1218	clinic	JNDC01000107	2,690	3	WP_013641249.1	66
Pseudomonas avellanae strain R25260	plant	MLEG01000115	4,896	6	POC98691.1	65
Acidithiobacillus thiooxidans A01	wastewater	AZMO01000115	9,773	12	WP_051488149.1	73
Acidithiobacillus thiooxidans strain JYC-17	wastewater	LWSD01000209	10,265	15	OCX85502.1	73
Acidiphilium sp.	wastewater	NCBH01000069	5,360	6	WP_083818739.1	66
Enterobacter cloacae strain GEO_49_Up_A	wastewater	QKOM01000204	2,797	3	RWS52735.1	82
Verminephrobacter eiseniae EF01-2	animal	NC_008771.1	31,194	43	WP_083758778.1	74
Acidiphilium multivorum AIU301	wastewater	NC_015187.1	65,564	68	WP_013641249.1	66

Supplementary Table 5. Oligonucleotides used in this study

**Name**	**DNA sequence (5' to 3')**
pBBR1_for	GCGGCCACCGGCTGGCT
pBBR1_rev	TACCGGCGCGGCAGCGTGACCC
nptII_gib_for	GCGCCGGTAGATCTGCTCATGTTTGAAGCTTCACGCTGCCGCA
nptII_gib_rev	CGGTGGCCGCCAAAAAGGCCATCCGTCAGGTCAGAAGAACTCGT
seq_pCON_var_for	CGACTGTGGCCGGCTGG
nptII_rev	TCAGAAGAACTCGTCAAGAAGGCG
pCON-var_gib_for	TTCTTGACGAGTTCTTCTGACAGCGGCCACCGGCTGGCTCGCTTC
pCON-var_gib_rev	GAGCCAGCCGGTGGCCGCTGTCAGAAGAACTCGTCAAGAAGGCGA
pCON-ran_gib_for	CTTGACGAGTTCTTCTGACAGGCTCATACCCCTGCCG
pCON-ran_gib_rev	CCAGCCGGTGGCCGCTGCGCATCGTGACGGCC
pBAD_gib_for	TCACGCTGCCGCGCCGGTAGTTATGACAACTTGACGGCTACATCATTC
pBAD_gib_rev	GCGAGCCAGCCGGTGGCCGCCAAAAAGGCCATCCGTCAGG
q_idnT_F	GTGGGTTTTGTCCTGCTGTT
q_idnT_R	ACAGAGAGCGCTGCTACCAT
q_nptII_F	GCTCCTGCCGAGAAAGTATC
q_nptII_R	TCATCCTGATCGACAAGACC
q_pBB_F	CACGAGGTGATGCAGCAG
q_pBB_R	GGCCACGCAGTCCAGAG
